# Federated Learning used for predicting outcomes in SARS-COV-2 patients

**DOI:** 10.21203/rs.3.rs-126892/v1

**Published:** 2021-01-08

**Authors:** Mona Flores, Ittai Dayan, Holger Roth, Aoxiao Zhong, Ahmed Harouni, Amilcare Gentili, Anas Abidin, Andrew Liu, Anthony Costa, Bradford Wood, Chien-Sung Tsai, Chih-Hung Wang, Chun-Nan Hsu, CK Lee, Colleen Ruan, Daguang Xu, Dufan Wu, Eddie Huang, Felipe Kitamura, Griffin Lacey, Gustavo César de Antônio Corradi, Hao-Hsin Shin, Hirofumi Obinata, Hui Ren, Jason Crane, Jesse Tetreault, Jiahui Guan, John Garrett, Jung Gil Park, Keith Dreyer, Krishna Juluru, Kristopher Kersten, Marcio Aloisio Bezerra Cavalcanti Rockenbach, Marius Linguraru, Masoom Haider, Meena AbdelMaseeh, Nicola Rieke, Pablo Damasceno, Pedro Mario Cruz e Silva, Pochuan Wang, Sheng Xu, Shuichi Kawano, Sira Sriswasdi, Soo Young Park, Thomas Grist, Varun Buch, Watsamon Jantarabenjakul, Weichung Wang, Won Young Tak, Xiang Li, Xihong Lin, Fred Kwon, Fiona Gilbert, Josh Kaggie, Quanzheng Li, Abood Quraini, Andrew Feng, Andrew Priest, Baris Turkbey, Benjamin Glicksberg, Bernardo Bizzo, Byung Seok Kim, Carlos Tor-Diez, Chia-Cheng Lee, Chia-Jung Hsu, Chin Lin, Chiu-Ling Lai, Christopher Hess, Colin Compas, Deepi Bhatia, Eric Oermann, Evan Leibovitz, Hisashi Sasaki, Hitoshi Mori, Isaac Yang, Jae Ho Sohn, Krishna Nand Keshava Murthy, Li-Chen Fu, Matheus Ribeiro Furtado de Mendonça, Mike Fralick, Min Kyu Kang, Mohammad Adil, Natalie Gangai, Peerapon Vateekul, Pierre Elnajjar, Sarah Hickman, Sharmila Majumdar, Shelley McLeod, Sheridan Reed, Stefan Graf, Stephanie Harmon, Tatsuya Kodama, Thanyawee Puthanakit, Tony Mazzulli, Vitor de Lima Lavor, Yothin Rakvongthai, Yu Rim Lee, Yuhong Wen

**Affiliations:** NVIDIA; MGH Radiology and Harvard Medical School; NVIDIA; Center for Advanced Medical Computing and Analysis, Department of Radiology, Massachusetts General Hospital, Harvard Medical School, Boston, MA; NVIDIA; San Diego VA Health Care System, San Diego; NVIDIA; Mount Sinai Health System; Radiology & Imaging Sciences / Clinical Center, National Institutes of Health; Division of Cardiovascular Surgery, Department of Surgery, Tri-Service General Hospital, National Defense Medical Center, Taipei, Taiwan, R.O.C.; Tri-Service General Hospital, National Defense Medical Center; Center for Research in Biological Systems, University of California, San Diego; NVIDIA; NVIDIA; NVIDIA; Center for Advanced Medical Computing and Analysis, Department of Radiology, Massachusetts General Hospital, Harvard Medical School, Boston, MA; NVIDIA; Diagnósticos da América SA (Dasa); NVIDIA; Memorial Sloan Kettering Cancer Center; Self-Defense Forces Central Hospital; Center for Advanced Medical Computing and Analysis, Department of Radiology, Massachusetts General Hospital, Harvard Medical School, Boston, MA; Center for Intelligent Imaging, Department of Radiology and Biomedical Imaging, University of California, San Francisco, California, USA.; NVIDIA; NVIDIA; The University of Wisconsin-Madison School of Medicine and Public Health; Yeungnam University College of Medicine; Center for Clinical Data Science, Massachusetts General Brigham, Boston, MA; Memorial Sloan Kettering Cancer Center; NVIDIA; Center for Clinical Data Science, Massachusetts General Brigham, Boston, MA; Sheikh Zayed Institute for Pediatric Surgical Innovation, Children’s National Hospital and School of Medicine and Health Sciences, George Washington University, Washington, DC; Joint Dept. of Medical Imaging, Sinai Health System, University of Toronto, Toronto, Canada and Lunenfeld-Tanenbaum Research Institute, Toronto, Canada; Lunenfeld-Tanenbaum Research Institute, Toronto, Canada; NVIDIA; Center for Intelligent Imaging, Department of Radiology and Biomedical Imaging, University of California, San Francisco, California, USA.; NVIDIA; MeDA Lab and Institute of Applied Mathematical Sciences, National Taiwan University, Taipei, Taiwan; Center for Interventional Oncology, National Institutes of Health, Bethesda, MD, USA; Self-Defense Forces Central Hospital; Chulalongkorn University; Department of Internal Medicine, School of Medicine, Kyungpook National University, Daegu, South Korea; University of Wisconsin-Madison; Center for Clinical Data Science, Massachusetts General Brigham, Boston, MA; Department of Pediatrics, Faculty of Medicine, Chulalongkorn University, Bangkok, Thailand and Thai Red Cross Emerging Infectious Diseases Clinical Center, King Chulalongkorn Memorial Hospital, Bang; National Taiwan University; Department of Internal Medicine, School of Medicine, Kyungpook National University, Daegu, South Korea; Center for Advanced Medical Computing and Analysis, Department of Radiology, Massachusetts General Hospital, Harvard Medical School, Boston, MA; Harvard T.H. Chan School of Public Health; Mount Sinai Health System; University of Cambridge; Department of Radiology, NIHR Cambridge Biomedical Resource Centre, University of Cambridge; Center for Advanced Medical Computing and Analysis, Department of Radiology, Massachusetts General Hospital, Harvard Medical School, Boston, MA; NVIDIA; NVIDIA; Department of Radiology, NIHR Cambridge Biomedical Resource Centre, Cambridge University Hospital; National Institutes of Health; Icahn School of Medicine at Mount Sinai; Center for Clinical Data Science, Massachusetts General Brigham, Boston, MA; Department of Internal Medicine, Catholic University of Daegu School of Medicine, Daegu, South Korea; Sheikh Zayed Institute for Pediatric Surgical Innovation, Children’s National Hospital, Washington, DC; Planning and Management Office, Tri-Service General Hospital, National Defense Medical Center, Taipei, Taiwan, R.O.C. and Division of Colorectal Surgery, Department of Surgery, Tri-Service General H; Planning and Management Office, Tri-Service General Hospital, National Defense Medical Center, Taipei, Taiwan, R.O.C.; School of Medicine, National Defense Medical Center, Taipei, Taiwan, R.O.C. and School of Public Health, National Defense Medical Center, Taipei, Taiwan, R.O.C. and Graduate Institute of Life Scienc; Medical Review and Pharmaceutical Benefits Division, National Health Insurance Administration, Taipei. Taiwan; University of California, San Francisco; NVIDIA; NVIDIA; NYU Langone; The Center for Clinical Data Science, Mass General Brigham.; Self-Defense Forces Central Hospital; Self-Defense Forces Central Hospital; NVIDIA; Center for Intelligent Imaging, Department of Radiology and Biomedical Imaging, University of California, San Francisco, California, USA.; Memorial Sloan Kettering Cancer Center; MOST/NTU All Vista Healthcare Center, Center for Artificial Intelligence and Advanced Robotics, National Taiwan University, Taipei, Taiwan; Diagnósticos da América SA (DASA); Division of General Internal Medicine and Geriatrics (Fralick), Sinai Health System, Toronto, Canada; Department of Internal Medicine, Yeungnam University College of Medicine, Daegu, South Korea; NVIDIA; Memorial Sloan Kettering Cancer Center; Department of Computer Engineering, Faculty of Engineering, Chulalongkorn University; Memorial Sloan Kettering Cancer Center; Department of Radiology, NIHR Cambridge Biomedical Resource Centre, University of Cambridge; Center for Intelligent Imaging, Department of Radiology and Biomedical Imaging, University of California, San Francisco, California, USA.; Schwartz/Reisman Emergency Medicine Institute, Sinai Health, Toronto, ON, Canada and Department of Family and Community Medicine, University of Toronto, Toronto, ON, Canada; Center for Interventional Oncology, National Institutes of Health, Bethesda, MD, USA; University of Cambridge; National Cancer Institute; Self-Defense Forces Central Hospital; Department of Pediatrics, Faculty of Medicine, Chulalongkorn University, Center of Excellence in Pediatric Infectious Diseases and Vaccine, Chulalongkorn University; Department of Microbiology, Sinai Health/University Health Network, Toronto, Canada and Department of Laboratory Medicine and Pathobiology, University of Toronto, Toronto. Canada Public Health Ontar; Diagnósticos da América SA (DASA); Chulalongkorn University Biomedical Imaging Group and Division of Nuclear Medicine, Department of Radiology, Faculty of Medicine, Chulalongkorn University, Bangkok, Thailand; Department of Internal Medicine, School of Medicine, Kyungpook National University, Daegu, South Korea; NVIDIA

**Keywords:** federated learning, artificial intelligence, SARS-COV-2

## Abstract

‘Federated Learning’ (FL) is a method to train Artificial Intelligence (AI) models with data from multiple sources while maintaining anonymity of the data thus removing many barriers to data sharing. During the SARS-COV-2 pandemic, 20 institutes collaborated on a healthcare FL study to predict future oxygen requirements of infected patients using inputs of vital signs, laboratory data, and chest x-rays, constituting the “EXAM” (EMR CXR AI Model) model. EXAM achieved an average Area Under the Curve (AUC) of over 0.92, an average improvement of 16%, and a 38% increase in generalisability over local models. The FL paradigm was successfully applied to facilitate a rapid data science collaboration without data exchange, resulting in a model that generalised across heterogeneous, unharmonized datasets. This provided the broader healthcare community with a validated model to respond to COVID-19 challenges, as well as set the stage for broader use of FL in healthcare.

The scientific and academic medical and data science communities have come together in the face of the pandemic crisis in order to rapidly assess novel paradigms in artificial intelligence that are rapid and secure, and potentially incentivize data sharing and model training and testing without the usual privacy and data ownership hurdles of conventional collaborations^1,2^. Healthcare providers, researchers and industry have pivoted their focus to address unmet and critical clinical needs created by the crisis, with remarkable results^3–6^. Clinical trial recruitment has been expedited and facilitated by national regulatory bodies and an international cooperative spirit^7–9^. The data analytics and artificial intelligence (AI) disciplines have always fostered open and collaborative approaches, embracing concepts such as open-source software, reproducible research, data repositories, and making anonymized datasets publicly available^10,11^. The pandemic has emphasized the need to expeditiously conduct data collaborations that empower the clinical and scientific communities when responding to rapidly evolving and widespread global challenges. Data sharing has ethical, regulatory and legal complexities that are underscored, and perhaps somewhat complicated by the recent entrance of large tech companies into the healthcare data world^12–15^.

A concrete example for these types of collaborations is our recent work on an AI-based SARS-COV-2 Clinical Decision Support (CDS) algorithm. The CDS predicts a risk score that can be used to support decisions to admit infected patients to the hospital and to help determine the level of hospital care they will likely require. We refined and validated the algorithm across multiple health systems. The CDS was created at Mass General Brigham (MGB), using chest x-ray (CXR) data, vital signs, demographic data, and lab values that were shown to be predictive of COVID-19 patient outcomes^16–18,16−19^. The CDS outputs a score, ‘CORISK’, that predicts oxygen support requirement, and can be used as a decision aid tool for triaging patients by front-line clinicians^20–22^.

Healthcare providers have preferred using algorithms that were validated on their own data^23^. To date, most AI algorithms have been trained and validated only on a few datasets that often lacked in diversity^24,25^, resulting in less generalisable performance. Even near-perfect peer-reviewed performance metrics do not guarantee generalisability nor a lack of over-fitting. Our aim was to develop an algorithm trained on a diverse dataset, making it useful, trusted and generalisable across a large number of healthcare systems. Accessing diverse data without the requirement of centralised data^26^ is enabled by techniques such as Transfer Learning^27^ and ‘Federated Learning’ (FL)^28^ for achieving distributed model training and validation. The authors chose FL due to its ability to rapidly launch centrally orchestrated experiments with improved traceability of data and assessment of algorithmic changes and impact^29^. FL has shown promise in recent medical imaging applications^30–33^, including COVID-19 analysis^34–37^, albeit with limited scale. Governance of data for FL is maintained locally, alleviating privacy concerns, with only model ‘weights’ or ‘gradients’ transferred between the client-sites and the federated server^38,39^.

Driven by the pandemic and enabled by the privacy-conserving nature of FL, 20 institutions were recruited, the majority of which were hospitals. The study named “EXAM” (**E**MR Chest **X**-Ray **A**I **M**odel), consisted of algorithm development by a Mass General Brigham team during March 2020, and the recruitment for this FL study that started in June. Between August and October, 140 experiments were conducted, and by end-October 2020, the refined version of the algorithm was made public on NVIDIA NGC^40^.

## A global dataset for COVID-19 image analysis

The 20 client-sites prepared 16,148 cases (both positive and negative) for the purpose of training, validating, and testing the model. Each case included one CXR and the requisite data inputs taken from the patient’s medical record. A breakdown of the cohort size of the dataset for each client site is shown in Fig. 1b. The significant diversity of data between sites motivated the researchers in creating the dataset, since capturing these differences was thought to be needed in order to create a performant CDS. The distribution and patterns of CXR image intensities (pixel values) varied significantly among the sites due to a multitude of patient and site-specific factors, such as differences in device manufacturers and imaging protocols, as shown in Fig. 1c. Patient age and EMR data varied for different sites due to the demographic differences between hospitals located around the globe (Fig. 1d and extended Data Fig. 1).

## An AI model to predict a ‘CORISK’ score

There is wide variation in the clinical course of patients who present to the hospital with symptoms of COVID-19, with some experiencing rapid deterioration in respiratory function requiring different interventions in order to prevent or mitigate hypoxemia^41,42^. A critical decision made during the evaluation of a patient at the initial point of care or the ED, is whether the patient is likely to require more invasive or resource-limited counter-measures or interventions (such as mechanical ventilation or monoclonal antibodies), and should therefore receive a scarce but effective therapy, a therapy with a narrow risk-benefit ratio due to side effects, or a higher level of care, such as admittance to the ICU^43,44^. In contrast, a patient who is at a lower risk of requiring invasive oxygen therapy may be placed in a less intensive care setting such as a regular ward or even released from the ED for continued self-monitoring at home^45^.

Therefore, the model was trained to predict the ‘CORISK’ score corresponding to a patient’s oxygen needs within two prediction windows, 24 hours and 72 hours after initial presentation to the ED. We set the outcome labels of patients as 0, 0.25, 0.5, and 0.75 if the most intensive oxygen therapy the patient received in the prediction window was room air (RA), low-flow oxygen (LFO), high-flow oxygen (HFO)/non-invasive ventilation (NIV), or mechanical ventilation (MV), respectively. If the patient died within the prediction window, the outcome label was set to 1. This resulted in each case being assigned two labels in the range of 0 to 1, corresponding to each of the prediction windows. For EMR features, data preprocessing included de-identification, missing value imputation (using the MissForest algorithm^46^), and normalization to zero-mean and unit variance. CXR images were preprocessed to select the right series and exclude lateral view images, then scaled to a resolution of 224 × 224. As shown in Fig. 2, the model fuses information from both the EMR features and CXR features (based on a modified ResNet-34 with spatial attention^47,48^ pre-trained on the CheXpert dataset)^49^, and Deep & Cross network^50^. In order to converge these different data types, a 512-dimensional feature vector was extracted from each CXR image using a pre-trained ResNet-34, with spatial attention, then concatenated with the EMR features as the input for the Deep & Cross network (see Methods). The final output was a continuous value from 0 to 1 for both the 24 hour and 72-hour predictions, corresponding to the labels described above. We used binary cross-entropy as the loss function and ‘Adam’ as the optimizer. The model was implemented in Tensorflow^51^ using the NVIDIA Clara Train SDK^52^. The average AUC for the three prediction tasks (LFO, HFO/NIV, or MV) was calculated and used as the final evaluation metric (see Methods).

## Performance boosts through Federated Learning

**Figure 3:**
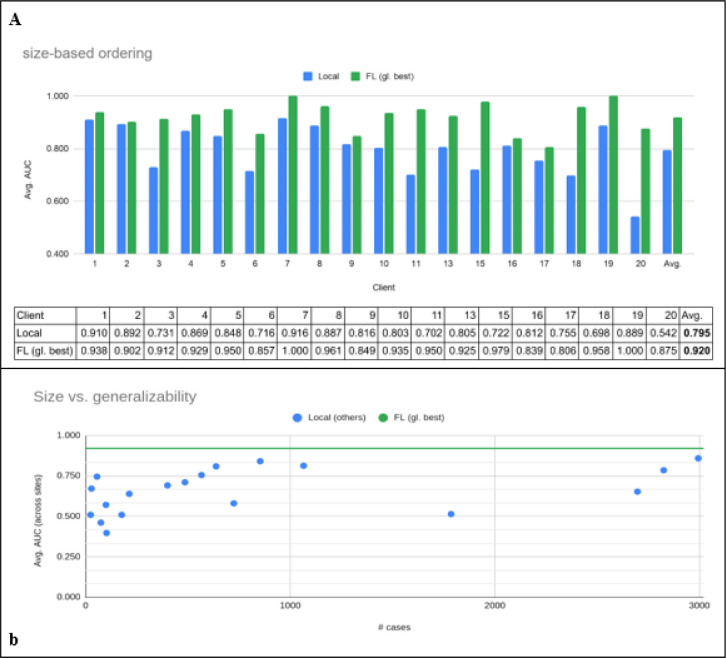
Federated Learning vs. local training performance. a, Test performance of models predicting 24h oxygen treatment trained on local data only (Local) versus the performance of the best global model available on the server (FL (gl. best)). b, Generalisability (average performance on other sites’ test data) as a function of a client’s dataset size (# cases). The average performance improved by 16% compared to locally trained models alone, while average generalisability of the global model improved by 38%. Note, we show the performance for 18 of 20 clients here as client 12 had only outcomes for 72 hours (see Extended Data Fig. 7) and client 14 only cases with room air treatment, resulting in the evaluation metric (avg. AUC) being not applicable (see Methods).

Arguably, the most established form of FL is implementing the *Federated Averaging* algorithm proposed by McMahan et al^53^, or variations thereof. This algorithm can be realised using a client-server setup, where each participating site acts as a client. One can think of FL as a method aiming to minimize a global loss function by reducing a set of local loss functions, which are estimated at each site. By minimizing each client site’s local loss while also synchronizing the learned client site weights on a centralized aggregation server, one can minimize the global loss without needing to access the entire dataset in a centralized location. Each client site learns locally, and shares model weight updates with a central server that aggregates contributions using secure SSL encryption and communication protocols^54^. The server then sends an updated set of weights to each client site after the aggregation, and sites resume training locally. The server and client site iterate back and forth until the model converges (see Methods section). To analyse the stability of these results, we repeated three runs of local training and FL on different randomized data splits. Training the model through FL resulted in a significant performance improvement (p < < 1e-3, Wilcoxon signed-rank test) of 16% (as defined by the average-AUC when running the model on respective local test sets) and a 38% generalisability improvement (as defined by the average-AUC when running the model on all test sets) of the final global model for predicting 24 h oxygen treatment compared to models trained only on a site’s own data (Fig. 3). The results for predicting 72 h oxygen treatments are shown in Extended Data Fig. 7 and resulted in a performance improvement of 18% compared to locally trained models alone, while generalisability of the global model improved by 34%.

## Security Considerations

A primary motivation for healthcare institutes to use FL is to preserve the security and privacy of their data, as well as adhere to data compliance measures. However, there remains a potential risk of model ‘inversion’^55^ or even reconstructing training images from the model gradients themselves^56^. To counter these risks, there are security-enhancing measures that may be able to mitigate risk in the event of data ‘interception’ during site-server communication^57^. We investigated a partial weight-sharing scheme^58,59^ showing that models can reach a comparable performance even when only 25% of the weight updates are shared (Fig. 4 and Methods section). The weight updates were ranked during each iteration by magnitude of contribution and only a certain percentage of the largest weight updates were shared with the server (see Methods). With this, we validated previous findings, showing that partial weight sharing, and other differential privacy techniques can successfully be applied in FL^58^.

## Impact on patient care

To our knowledge, this study features the largest real-world healthcare FL experiment to date in terms of number of sites and number of data points used. The study encompassed 20 client-sites and included over 16,000 cases (Extended Data Table 2). We believe that it provides a powerful case study for the utilization of FL involving multiple sites across 5 continents and under the supervision of different regulatory bodies. The global algorithm proved to be more robust and achieved better results on individual sites than any model that was trained on local data. We believe that the consistent improvement was achieved not only due to a larger, but also a more diverse data set.

We observed that FL improved the prediction accuracy on all site testing sets, even when sites had relatively large local training data sets. For sites with small datasets, it was virtually impossible to build a performant deep learning model using only their local data. Furthermore, sites whose local models were trained with unbalanced cohorts (e.g., with most subjects experiencing mild cases of COVID-19) markedly benefited from the FL approach (Extended Data Figs. 3 & 4). More importantly, the generalisability of the FL model increased considerably, over the locally trained model, most likely since a population or an age group that are under-represented in one hospital/region could be highly represented in another region (Extended Data Figs. 5 & 6 and Extended Data Table 3). For example, children might be differentially affected by COVID-19, including their manifestations in lung imaging^60^.

As seen in Fig. 1c/d and Extended Data Fig. 1, we designed our study to resemble real-life clinical situations by intentionally not completing a meticulous harmonization of the data inputs. The features derived from the medical record were carefully defined in order to mitigate potential biases (Extended Data Table 1). Features that were expected to be influenced by different clinical practices and standards of care were avoided, such as reported symptoms or clinical impressions. We also chose model outputs that we believed to be objective outcomes which are fairly practical to discern, being low-flow oxygen treatment, high-flow oxygen treatment, mechanical ventilation, and death (Extended Data Fig. 2). We believe that these design considerations played a significant part in increasing the benefits from a FL approach and its impact on model performance, generalisability, and ultimately, its usability. By participating in this study, the client-sites received access to an optimized AI model (‘global FL model’), that can be further validated ahead of introduction into clinical care. The client-sites did not transfer data to a central repository but rather created a distributed data framework that can facilitate ongoing collaboration on AI model development and validation. We believe that the preservation of privacy, afforded by FL, encouraged participation of institutes who recognized the urgency to contribute during the COVID pandemic, and were not held back by data governance constraints. As mentioned above, we also experimented with techniques to avoid ‘interception’ of FL data, and found them to be promising (Fig. 4). This is an added security feature that we believe will encourage more institutions to use FL.

## Future development and outlook

In the opinion of this group, the main areas for development arising out of this collaboration will be to streamline data access, preparation and methods in order to better leverage a network of sites participating in FL. A system that would allow real-time model inference and processing would also be of benefit and would ‘close the loop’ from training to model deployment. Patient cohort identification and data harmonization are not new issues in research and data science^61^, but are further complicated given the lack of visibility on other sites’ data sets associated with FL. There is also a need for evolving our understanding of architectural considerations that will enable capturing more value out of FL, e.g., explicitly addressing the data domain shifts between the different participating sites^62^. Hyperparameter engineering can allow algorithms to ‘learn’ more effectively from larger data batches and adapt model parameters to a particular site for further personalization. For example, socio-economic status or ethnicity in an algorithm prototyped on a homogenous population could enable algorithms to capture more diversity in FL training, despite being less meaningful when only leveraging a single-site data set. Additionally, there is a need to improve our ability to predict each client-site’s contribution to improving the global FL model, which will help in client-site selection and prioritizing data acquisition and annotation efforts in the future. The latter is especially important given the high costs and difficult logistics of these large consortia endeavors, and the opportunity to capture diversity rather than sheer quantity of data samples.

## Methods

### Ethics approval

All procedures were conducted in accordance with principles for human experimentation as defined in the Declaration of Helsinki and International Conference on Harmonization Good Clinical Practice guidelines and approved by the relevant institutional review boards (e.g., the Mass General Brigham ethics board, reference # 2020P002673). Since no patient data was transferred between any of the participants and the study was considered of minimal risk to patients, the requirement of a full IRB process was largely waived according to the Ethical Principles and Guidelines for the Protection of Human Subjects of Research (the “Belmont Report”) and the requirements of the Health Insurance Portability and Accountability Act (HIPAA) of 1996.

### Data collection details

The cohorts for this study consisted of patients who presented to the Emergency Department with symptoms suspicious for COVID at the participating institutions:

Mass Gen Brigham affiliated hospitals (Mass General Hospital, Brigham and Women’s Hospital, Newton-Wellesley Hospital, North Shore Medical Center, Faulkner Hospital); Children’s National Hospital in Washington, D.C.; NIHR Cambridge Biomedical Research Centre; The Self-Defense Forces Central Hospital in Tokyo; National Taiwan University MeDA Lab and MAHC and Taiwan National Health Insurance Administration; Tri-Service General Hospital in Taiwan; Kyungpook National University Hospital in South Korea; Faculty of Medicine, Chulalongkorn University in Thailand; Diagnosticos da America SA in Brazil; University of California, San Francisco; VA San Diego; University of Toronto; National Institutes of Health in Bethesda, Maryland; University of Wisconsin-Madison School of Medicine and Public Health; Memorial Sloan Kettering Cancer Center in New York; and Mount Sinai Health System in New York.

The inclusion criteria were: 1. patient presented to the hospital’s Emergency Department (ED) or equivalent, 2. patient had a PCR test done during the current hospitalization or had a COVID PCR test with a positive result prior to hospitalization, 3. patient had a CXR in the ED or during the hospital stay, 4. Patient’s record had at least 5 of the EMR values (vitals, lab results and outcomes) detailed in Extended Data Table 1 obtained in the ED or during hospitalization.

The CXR and the EMR features used were the first available CXR and EMR values available for each patient obtained during this hospital stay. The datasets included COVID positive and COVID negative patients, determined by the PCR test. Client sites included all of their patients with a PCR positive test. Since most had more COVID negative than positive patients, we limited the number of negative patients included to at most 95% of the total cases at each client-site. In total, 21 EMR features were used as input to the model. The outcome (i.e., “ground truth”) labels were assigned based on patient requirements after 24- and 72-hour periods from initial admission to the ED. A detailed list of the requested EMR features and outcomes can be seen in Extended Data Table 1.

The variation of these features across different client-sites can be appreciated in Extended Data Fig.1. Data harmonization was not performed between different client-sites in order to train a robust model that could generalise well to unseen patient populations.

The distribution of oxygen treatment using different devices at different client-sites is shown in Extended Data Fig.2, which details the device usage at admission to the Emergency Department (ED), and after 24-hour and 72-hour periods.

The number of positive COVID-19 cases, confirmed by a single PCR test, are listed in Extended Data Table 2. Each client-site was asked to randomly split its dataset into 3 parts, 70% for training, 10% for validation, and 20% for testing. The random splits were generated independently for each of the repeated three local and FL training and evaluation experiments for both 24h and 72h outcome prediction models.

### Feature imputation & normalization

A MissForest algorithm^1^ was used to impute EMR features, based on the local training dataset. If an EMR feature was completely missing from a client-site dataset, the mean value of that feature, calculated exclusively on data from MGB client-sites, was used. Then, EMR features were rescaled to zero-mean and unit-variance based on statistics calculated on data from the MGB client-sites.

### Details of the EMR-CXR data fusion

To model the interactions of features from EMR and CXR data on a case-level, a deep feature scheme was used, based on Deep & Cross network architecture^2^. Binary/categorical features for the EMR inputs, as well as 512-dimensional image features in the CXR, were transformed into fused dense vectors of real values by embedding and stacking layers. The transformed dense vectors served as input to the fusion framework, which specifically employed a crossing network to enforce fusion among input from different sources. The crossing network performed explicit feature crossing within its layers, by conducting inner products between the original input feature and output from the previous layer, thus increasing the degree of interaction across features. At the same time, two individual classic deep neural networks with several stacked fully-connected feed-forward layers were trained. The final output of our framework was then derived from the concatenation of both classic and crossing networks.

### CORISK model and derivation of clinical score

Our preliminary, single-site patient outcome prediction model (calculating a risk score termed as “CORISK”) was trained using the MGB COVID cohort consisting of over 7,000 patients with a positive or undetermined COVID status (at time of data collection). EMR data and CXR images of these patients were extracted from the Enterprise Data Warehouse (EDW) and clinical Picture Archiving and Communication System (PACS) systems during the period extending from March to May 2020. The CORISK model was validated using data from five hospitals within the MGB system, and cross-validated using different time periods during the study period. It achieved an average prediction accuracy of over 85%. We further derived the clinical scores and the corresponding diagnostic criteria (“CORISK24” and “CORISK72”, for 24- and 72-hours patient outcome assessment), similar to CORISK model’s predictions. The clinical scores could be used by clinicians to triage patients into appropriate care settings.

The evaluation of the model is based on the average AUC of three prediction tasks derived from the CORISK score (LFO, HFO/NIV or MV). To compute it, we generate three sets of labels and predictions *L1* = {*P*_*pred*_*,P*_*gt*_}^3^ 0.25, *L2*={*P*_*pred*_*,P*_*gt*_}^3^ 0.5, and *L3* = {*P*_*pred*_*,P*_*gt*_}^3^ 0.5, where *P*_*pred*_ is the models CORISK predictions and *P*_*gt*_ is the ground truth CORISK scores representing a specific oxygen treatment as described above for a client-site’s test set. The average AUC was then computed as AUC =1/3 * (auc(*L1*) + auc*(L2*) + auc(*L3*)).

### Federated learning details

A pseudo-algorithm of FL is shown in Extended Data Algorithm 1. In our experiments, we set the number of federated rounds to be *T*=200, with one local training epoch per round t at each client. The number of clients *K* was up to 20, depending on the network connectivity of clients or available data for a specific targeted outcome period (24h or 72h). The number of local training iterations nk depends on the dataset size at each client *k* and is used to weigh each client’s contributions when aggregating the model weights in *FederatedAveraging*. During FL, each client-site selects its best local model by tracking the model’s performance on its local validation set. At the same time, the server determines the best global model based on the average validation scores sent from each client-site to the server after each FL round. After the FL training finishes, the best local models and best global model are automatically shared with all client-sites and evaluated on their local test data.

When training on local data only (the baseline), we set the epoch number to 200. The Adam optimizer was used for both local training and FL with an initial learning rate of 5e-5 and a stepwise learning rate decay with a factor 0.5 after every 40 epochs, which is important for the convergence of *FederatedAveraging*^3^. Random affine transformations, including rotation, translations, shear, scaling, and random intensity noise and shifts were applied to the images for data augmentation during training.

Due to the sensitivity of batch normalization (BN) layers^4^ when dealing with different clients in a non-independent and identically distributed (non-IID) setting^5^, we found the best model performance to occur when keeping the pre-trained ResNet34 with spatial attention^6^ parameters fixed during FL (i.e. using a learning rate of zero for those layers). The Deep & Cross network that combines image features with the EMR features does not contain BN layers and hence was not affected by BN’s instability issues.

In this study, we investigated a privacy-preserving scheme that shares only partial model updates between server and client-sites. To be exact, the weight updates (aka. gradients) were shared only if their absolute value was above a certain percentile threshold t_k_^(t)^ (Fig. 4), which was computed from all non-zero gradients DWk(t) and could be different for each client *k* in each FL round *t*. Variations of this scheme could include additional clipping of large gradients or differential privacy schemes^7^ that add random noise to the gradients or even to the raw data before feeding it to the network^7,8^.

### Statistical analysis

We conducted a Wilcoxon signed-rank test to confirm the significance of the observed improvement in performance between the locally trained model and the FL model for the 24 and 72 hr time point (see Fig. 3 and Extended Data Fig. 6). The null hypothesis was rejected with a one-sided p-value << 1e-3 in both cases.

A Pearson’s correlation was used to assess the generalisability (robustness to other client-sites’ test data) of locally trained models in relation to respective local dataset size. Only a moderate correlation was observed (r=0.43, p=0.035, df = 17 for the 24h model and r=0.62, p=0.003, df=16 for the 72h model). This indicates that dataset size alone is not the only factor in determining a model’s robustness to unseen data.

To compare the ROC curves from different sites and FL global one (shown in Fig. 5), we bootstrapped 1000 replicates from the data and computed their AUCs. We standardized the difference D=(AUC1-AUC2)/s, where s is the standard deviation of the bootstrap differences and AUC1 and AUC2 the AUC of the two (original) ROC curves. By comparing D with normal distribution, we obtained the significance p-values illustrated in Table 4. With alternative hypotheses to be FL greater than the compared one, most of the p-values give very small values, indicating the statistical significance of FL outcomes. Computation of p-values was conducted in R with the pROC library^9^.

### Benefits to client-sites with small datasets

We compared locally trained models with the global FL model on each client’s test data. For a client-site with a relatively small dataset, there are two typical ways to get a model: one is to train locally with its own data, the other is to apply a model trained on a larger dataset. It is shown in Extended Data Fig. 5 that these two ways are outperformed on all three tasks by the FL model significantly, indicating that the benefit for client-sites with small datasets is huge.

Another particular site (client-site 16) had an unbalanced dataset, with most subjects being of mild disease severity and with only a few severe cases. Thus, the improvement in prediction accuracy for the category with few cases was substantial; see Extended Data Fig. 3, t >= 0.5 (categories >= high-flow oxygen device). The FL model achieved a higher *true positive rate* for the two positive (severe) cases at a markedly lower *false positive rate* compared to the local model, both shown in the receiver operating characteristic (ROC) plots and confusion matrices. The difference in dataset distribution for the two compared client-sites can be seen in Extended Data Fig. 4.

### Effect of different demographics

To investigate the effectiveness of our model on patients with different demographics, especially with different races, we test our model on the test set of 5 client-sites in the Boston area and show results for different race populations accordingly. The results of Black or African American and White or Caucasian population (We don’t show results for other races here due to the limited sample sizes) is shown in the Extended Data Table 3. We show the mean and the standard deviation of AUCs of the 5 local models and the AUC for the federated trained model on 3 tasks for both 24- and 72-h prediction. We can see that the improvement brought by federated training is consistent across different races.

### Effect of different COVID-19 status

Extended Data Fig. 6 shows the performance of our model in predicting oxygen treatment in 24/72h for COVID positive/negative patients respectively. The COVID status is determined by the PCR tests performed at the visit of ED. It can be shown that our model is robust to both COVID positive and negative patients. This is crucial for our model to be applied on all the patients to support their triage since the PCR test results are usually not available at the time of ED disposition.

### Limitations and areas for future research

The study found the global models (see under ‘Federated Learning Details’) to be more robust compared to locally trained models when assessed across all client-sites’ test data. Locally optimized models might provide improved performance on a client-site’s own test data, but usually resulted in a loss of generalisability. Local model selection always depends on the local validation set’s quality and how well it represents the real test data’s characteristics. In contrast, the global model selected based on the averaged validation scores from each client-site turns out to have better generalisability.

It is possible to achieve higher-performing models on a local dataset when tuning the training strategies more exhaustively^10^, such as varying data augmentation, learning rate schedule, and data sampling methods. However, generalisability to other sites’ data is still expected to be limited due to the lack of representative training data. Future approaches may incorporate automated hyperparameter searching^10^, neural architecture search^11^, and other automated machine learning (AutoML)^12^ approaches to find the optimal training parameters for each client-site more efficiently.

Slow or interrupted internet connectivity sometimes caused some clients’ model updates to be not included in each round of FL training. Such clients are commonly known as “stragglers”^13^. Future implementations of FL might specifically address this issue by allowing asynchronous updates^14^.

Known issues of BN in FL^4^ motivated us to fix our base model for image feature extraction^6^ in order to reduce the divergence between unbalanced client-sites. Future work might explore different types of normalization techniques in order to allow the training of AI models in FL more effectively when the clients’ data is non-IID.

Although privacy is a key concern for participants of FL, the actual quantification of data leakage during model training is still rather unexplored as most efforts revolve around IT security for the communication between participants and server. Future work could aim to quantify the amount of data leakage that is still recoverable by model inversion methods or attacks on the gradients. A quantifiable way to measure privacy would allow better choices for deciding the minimal privacy parameters necessary while maintaining clinically acceptable performance^7,8,15^.

A final, but important limitation to all machine learning models is that they are limited by the quality of the training data. Institutions interested in deploying these algorithms for clinical care need to understand the inherent biases in the training. For example, the ground truth data used in the training of the EXAM model was 24- and 72- hour oxygen consumption in the patient. It is assumed that the oxygen consumption is the oxygen need. However, in the early period of the COVID-19 pandemic, many patients were provided high flow oxygen prophylactically, regardless of their oxygen need. Such clinical practice could skew the oxygen need predictions made by this model.

## Supplementary Material

Supplement
