# Supplementary material for: Federated Learning used for predicting outcomes in SARS-COV-2 patients
Source: Res Sq. 2021 Jan 8:rs.3.rs-126892. Preprint. [Version 1] doi: 10.21203/rs.3.rs-126892/v1 (PMC7805458; doi:10.21203/rs.3.rs-126892/v1)

**Extended data figures and tables**

| 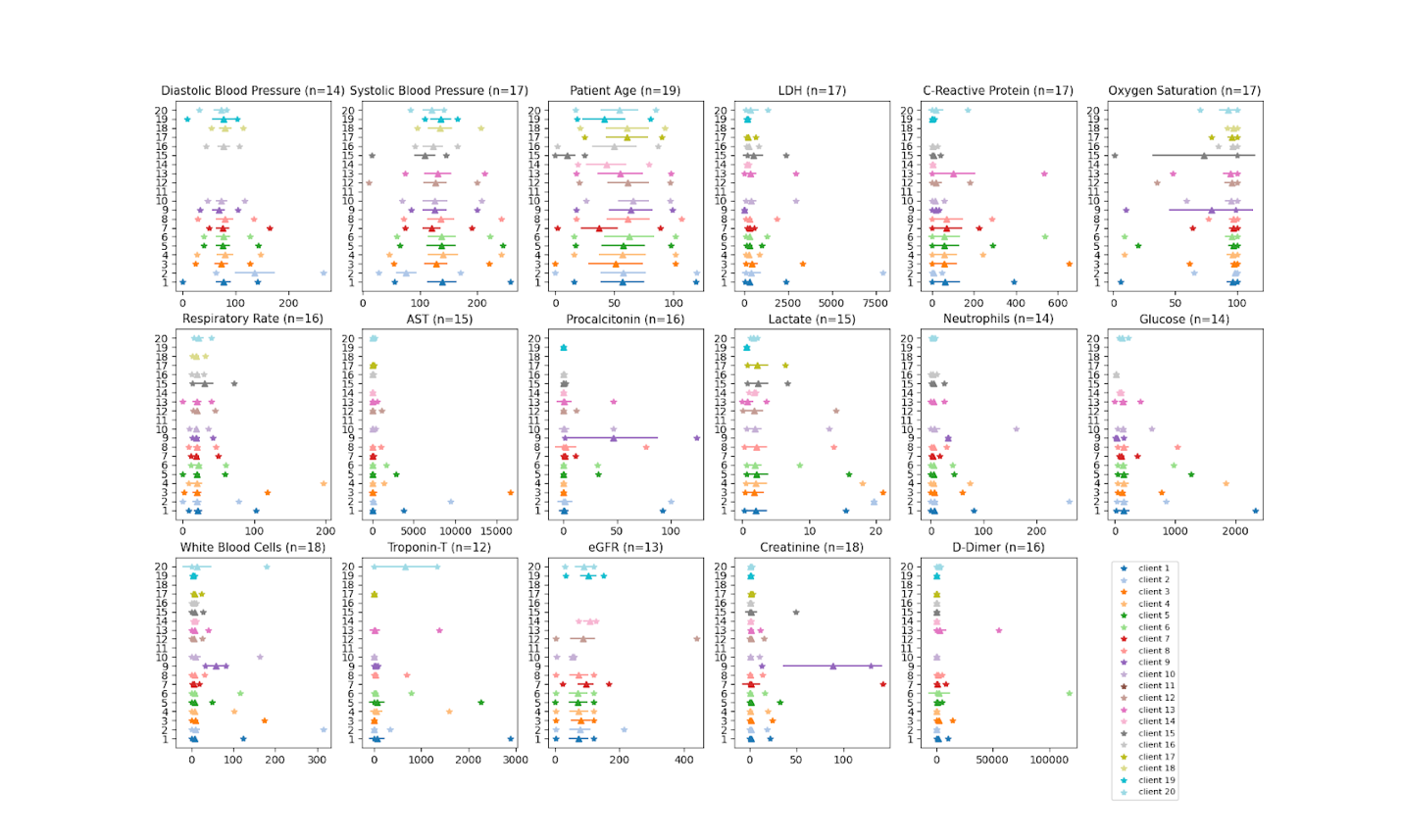 |
| --- |

**Extended Data** [**Fig.**](https://docs.google.com/document/d/1Wg4Qk0qxnWPjlK0IU5LicTAJ4wZjVCOAtr6BYasQ51U/edit?ts=5fc43130&pli=1#figur_emr)**1 | Characteristics of EMR data used in EXAM.** Min. and max. values (asterisks) and mean and standard deviation (length of bars) for each EMR feature used as an input to the model. ***n*** specifies the number of sites that had this particular feature available. Missing values were imputed using a MissedForest algorithm.

| 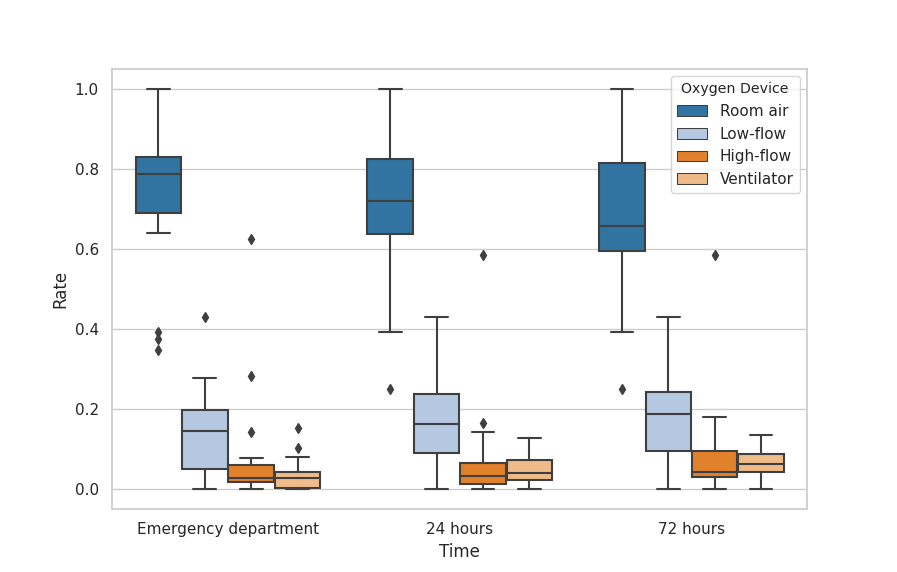 |
| --- |

**Extended Data** [**Fig.**](https://docs.google.com/document/d/1Wg4Qk0qxnWPjlK0IU5LicTAJ4wZjVCOAtr6BYasQ51U/edit?ts=5fc43130&pli=1#figur_oxygen)**2 | Distribution of oxygen treatments between EXAM sites.** The boxplots show the quartiles of the minimum, the maximum, the sample median, and the first and third quartiles (excluding outliers) of the oxygen treatments applied at different sites at time of emergency department admission and after 24 and 72- hour periods. The types of oxygen treatments administered are ‘room air’, ‘low-flow oxygen’, ‘high-flow oxygen (non-invasive)’, and ‘ventilator’.

| **a**  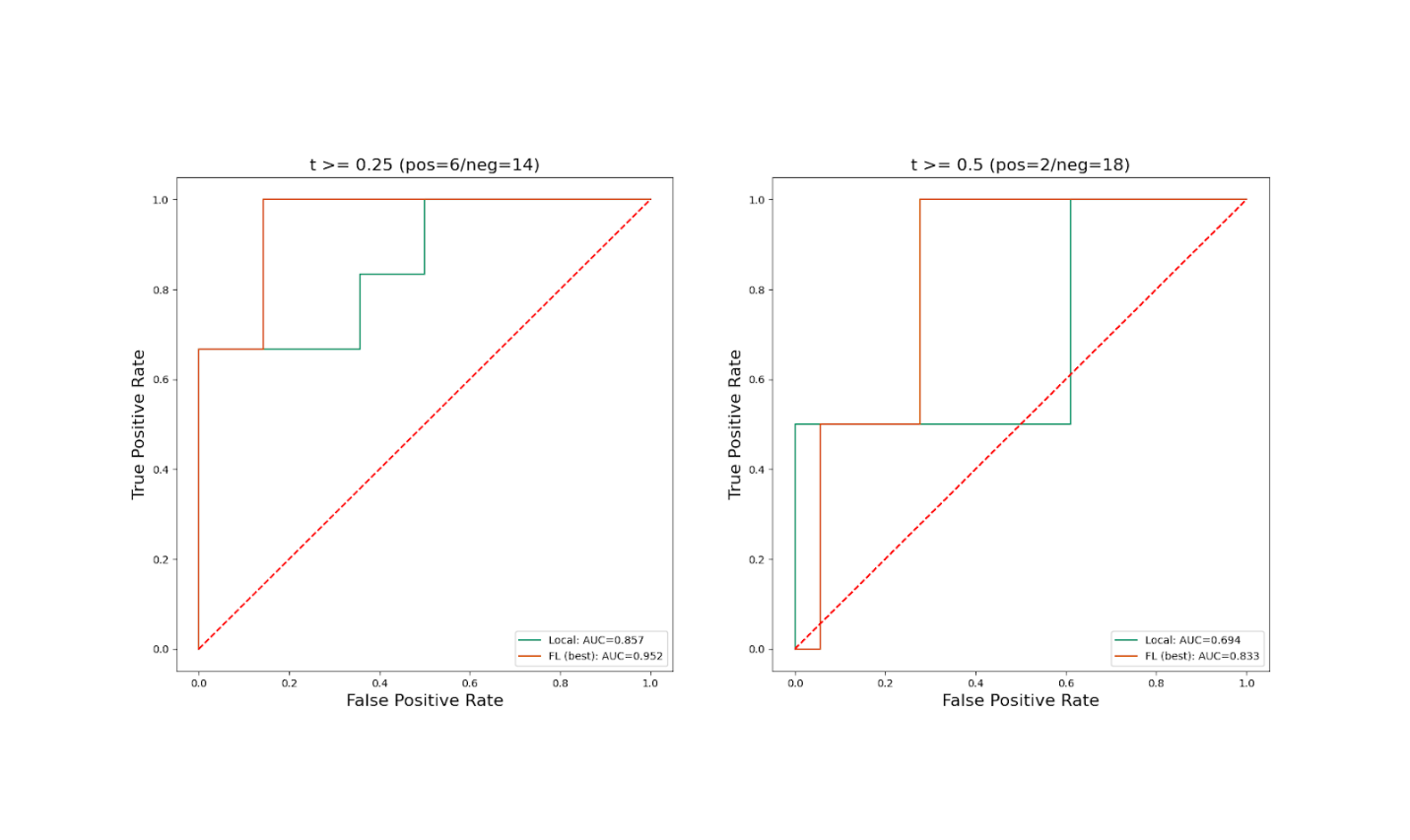 |
| --- |
| **b**  **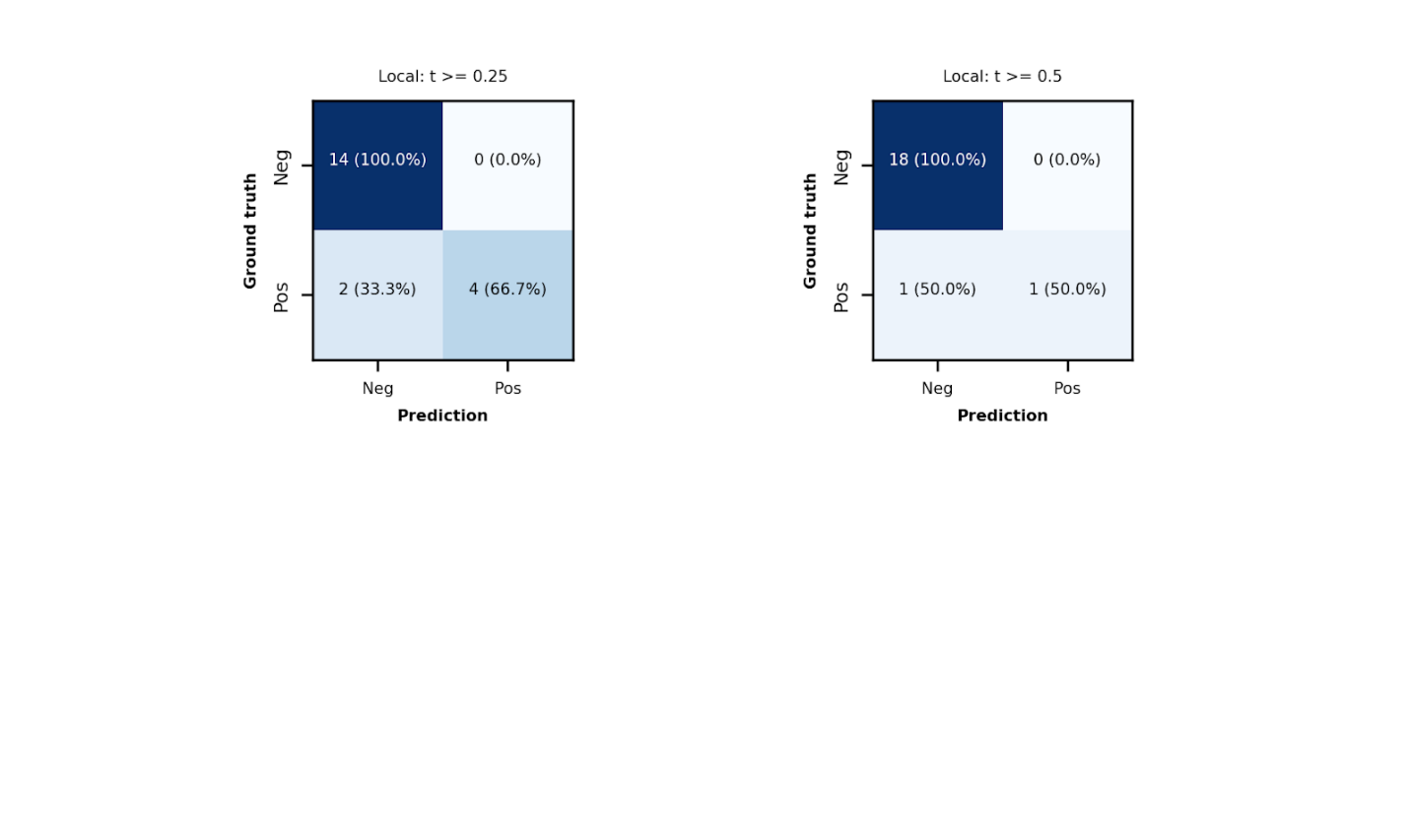** |
| **C**  **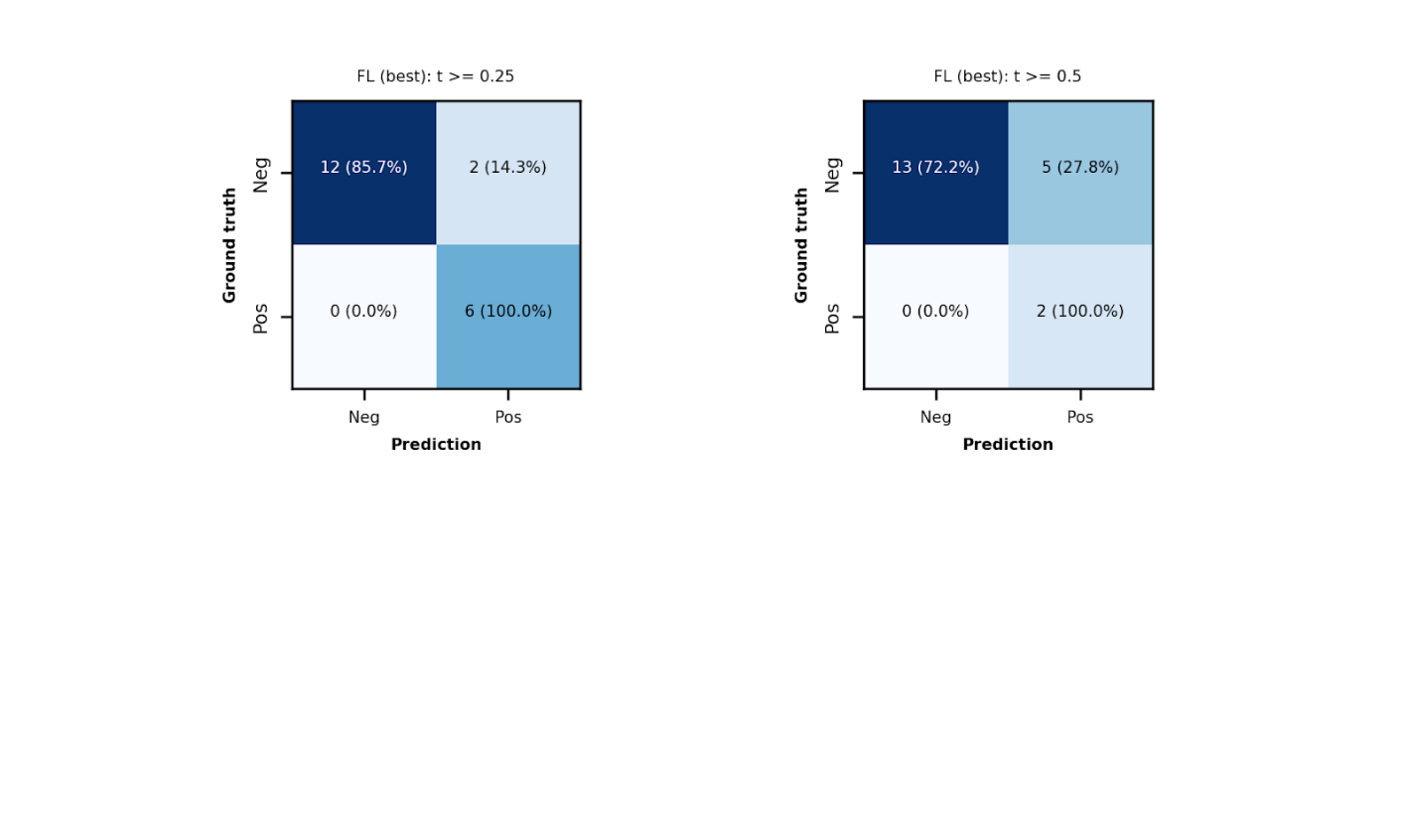** |

**Extended Data** [**Fig.**](https://docs.google.com/document/d/1Wg4Qk0qxnWPjlK0IU5LicTAJ4wZjVCOAtr6BYasQ51U/edit?ts=5fc43130&pli=1#figur_roc)**3 | Comparison of the Federated Learning trained vs. locally trained model at a site with unbalanced data and mostly mild cases.** a, ROC Performance. b, Confusion matrices on the test data at site 16 predicting oxygen treatment at 72h using the locally trained model. c, Confusion matrices on the test data at site 16 predicting oxygen treatment at 72h using the best Federated Learning global model.

| 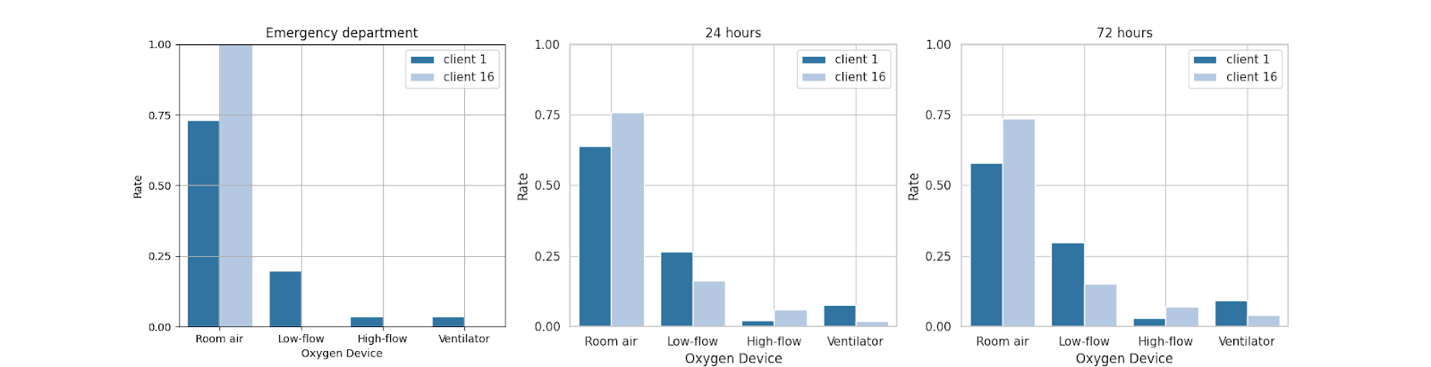 |
| --- |

**Extended Data** [**Fig.**](https://docs.google.com/document/d/1Wg4Qk0qxnWPjlK0IU5LicTAJ4wZjVCOAtr6BYasQ51U/edit?ts=5fc43130&pli=1#figur_clientsod)**4 | Site variations in oxygen usage.** Normalized distributions of oxygen devices at different time points, comparing the site with largest dataset size (site 1) and a site with unbalanced data, including mostly mild cases (site 16).


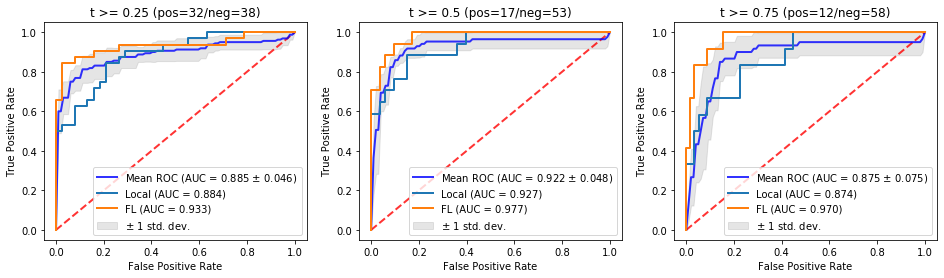


**Extended Data** [**Fig.**](https://docs.google.com/document/d/1Wg4Qk0qxnWPjlK0IU5LicTAJ4wZjVCOAtr6BYasQ51U/edit?ts=5fc43130&pli=1#figur_client12_roc)**5| Effect of small data set size on ROC**. ROC of the locally trained model and the mean ROC of models trained on larger datasets in comparison to the best global model to predict oxygen treatment at 72h, using the test data at site 12 - a site with relatively small dataset. The Mean ROC is calculated based on models trained on 5 large datasets from sites in the Boston area, with the gray-area showing the standard deviation of the ROCs.

24h Prediction for COVID positive patients


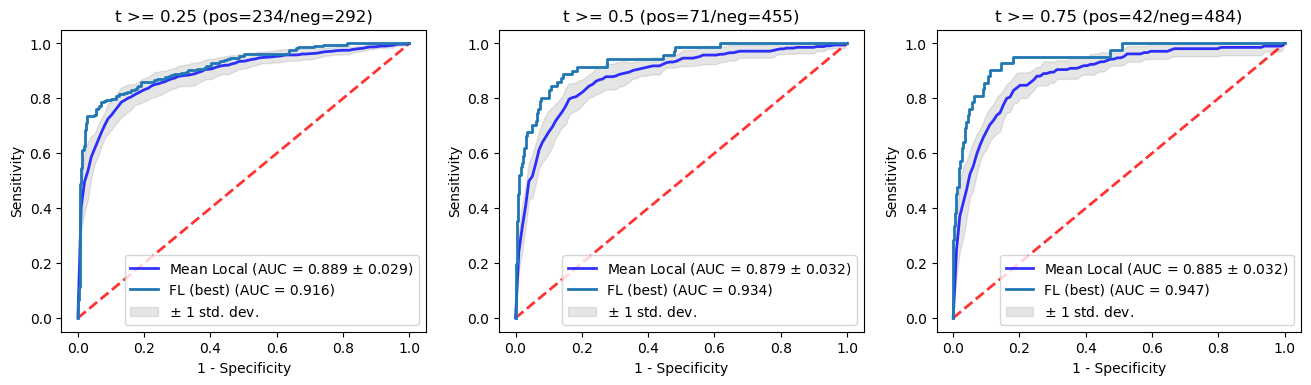


24h Prediction for COVID negative patients


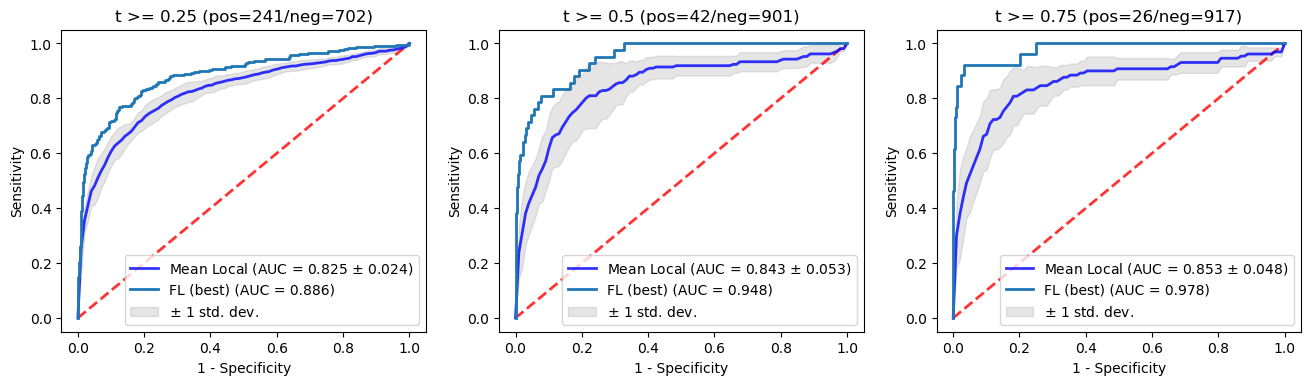


72h Prediction for COVID positive patients


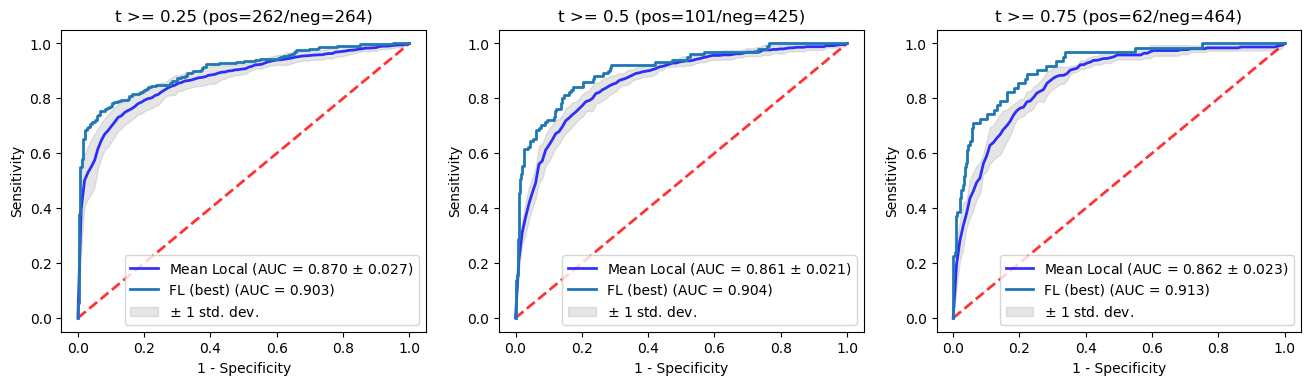


72h Prediction for COVID negative patients


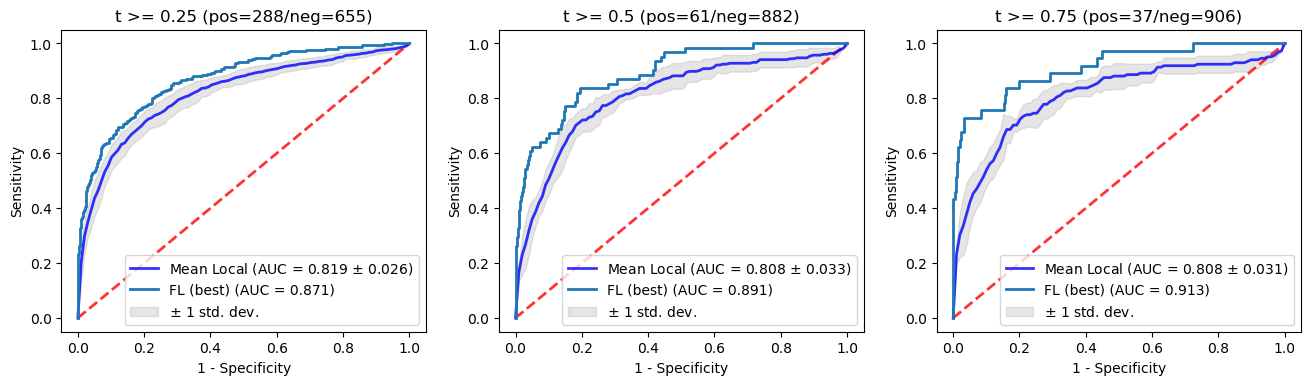


**Extended Data** [**Fig.**](https://docs.google.com/document/d/1Wg4Qk0qxnWPjlK0IU5LicTAJ4wZjVCOAtr6BYasQ51U/edit?ts=5fc43130&pli=1#figur_client12_roc)**6| Effect of large data set size on ROC.** ROCs of the best global model in comparison to the mean ROCs of models trained on local datasets to predict 24-/72-h oxygen treatment devices for COVID positive/negative patients respectively, using the test data of 5 large datasets from sites in the Boston area. The Mean ROC is calculated based on 5 locally trained models, with the gray-area showing the standard deviation of the ROCs.

| **a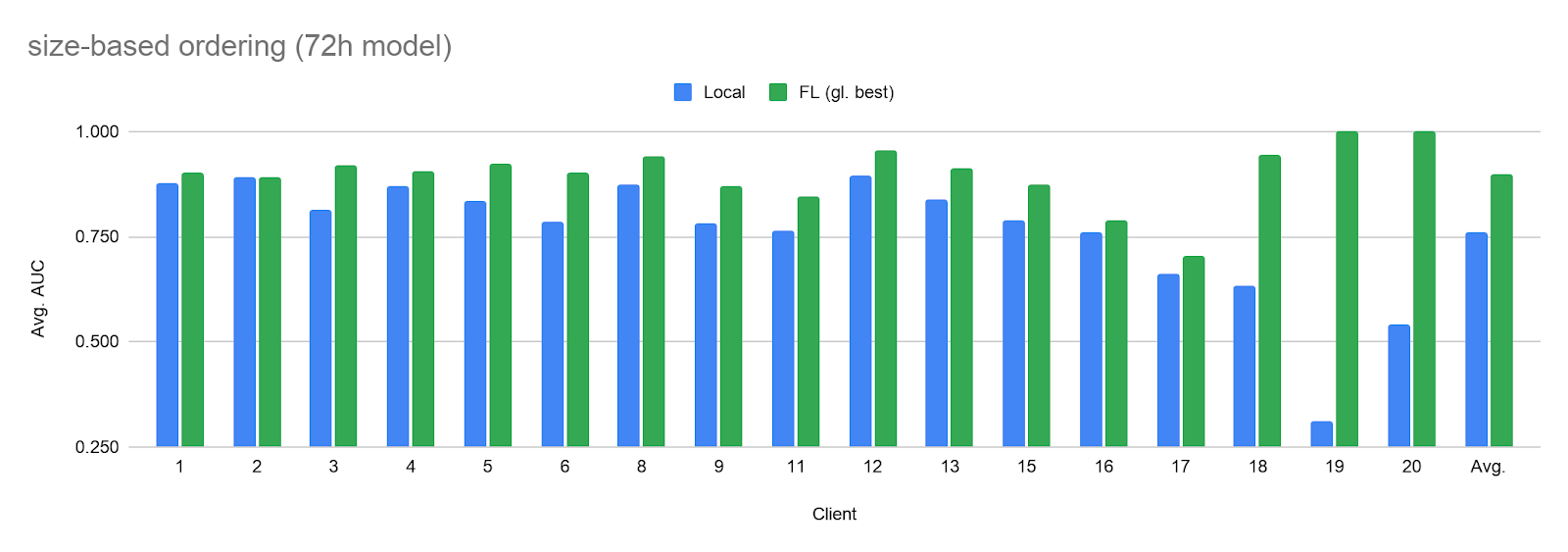**  **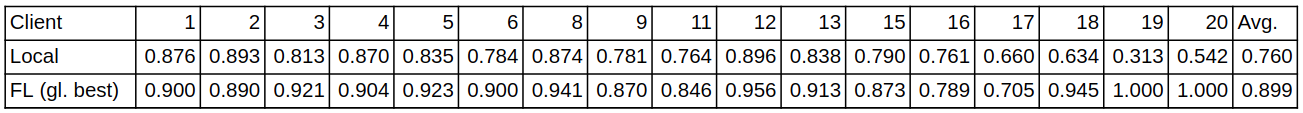** |
| --- |
| **b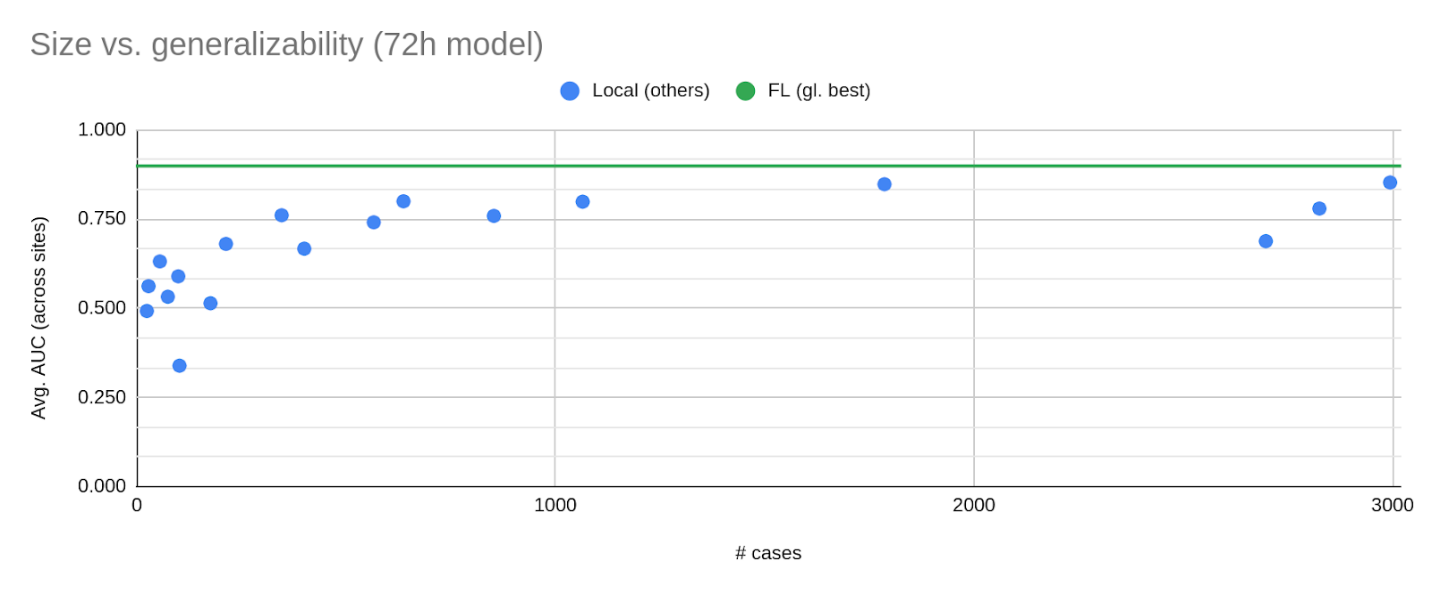** |

**Extended Data** Fig. **7 |** Test performance of models predicting 72h oxygen treatment trained on local data only (Local) versus the performance of the best global model available on the server (FL (gl. best)). **b,** Generalisability (average performance on other sites’ test data) as a function of a site’s dataset size (# cases). The average performance improved by 18% compared to locally trained models alone, while average generalisability of the global model improved by 34%.

**Extended Data** [**Table 1**](https://docs.google.com/document/d/1Wg4Qk0qxnWPjlK0IU5LicTAJ4wZjVCOAtr6BYasQ51U/edit?ts=5fc43130&pli=1#table_emr) **| EMR (electronic medical record) data used in the EXAM study**

| **Category** | **Subcategory** | **Component Name** | **Description** | **Units** | **LOINC Code** |
| --- | --- | --- | --- | --- | --- |
| Demographic | - | Patient Age | - | Years | 30525-0 |
| Imaging | Portable Chest X-Ray | - | AP or PA Portable Chest X-ray | - | 36554-4 |
| Lab Value | C-Reactive Protein | C Reactive Protein | Blood C-Reactive Protein Concentration | mg/L | 1988-5 |
| Lab Value | CBC (Complete Blood Count) | Neutrophils | Blood Absolute Neutrophils | 10^9/L | 751-8 |
| Lab Value | CBC (Complete Blood Count) | White Blood Cells | Blood White Blood Cell Count | 10^9/L | 33256-9 |
| Lab Value | D-Dimer | D-Dimer | Blood D-Dimer Concentration | ng/mL | 7799-0 |
| Lab Value | Lactate | Lactate | Blood Lactate Concentration | mmol/L | 2524-7 |
| Lab Value | LDH (Lactate Dehydrogenase) | LDH | Blood Lactate Dehydrogenase Concentration | U/L | 2532-0 |
| Lab Value | Metabolic Panel | Creatinine | Blood Creatinine Concentration | mg/dL | 2160-0 |
| Lab Value | Procalcitonin | Procalcitonin | Blood Procalcitonin Concentration | ng/mL | 33959-8 |
| Lab Value | Metabolic Panel | eGFR | Estimated Glomerular Filtration Rate | mL/min/1.73m2 | 69405-9 |
| Lab Value | Troponin | Troponin-T | Blood Troponin Concentration | ng/ml | 67151-1 |
| Lab Value | Hepatic Panel | AST | Blood AST Concentration | IU/L | 1920-8 |
| Lab Value | Metabolic Panel | Glucose | Blood Glucose Concentration | mg/dL | 2345-7 |
| Vital Sign | - | Oxygen Saturation | Oxygen Saturation | % | 59408-5 |
| Vital Sign | - | Systolic Blood Pressure | Systolic Blood Pressure | mmHg | 8480-6 |
| Vital Sign | - | Diastolic Blood Pressure | Diastolic Blood Pressure | mmHg | 8462-4 |
| Vital Sign | - | Respiratory Rate | Respiratory Rate | breaths per minute | 9279-1 |
| Vital Sign |  | COVID PCR test | PCR for RNA [not used as input to model] |  | 95425-5 |
| Vital Sign | Oxygen Device used at ED (Emergency Department) | Oxygen Device | Ventilation, High-flow/NIV, Low-flow, Room Air | - | 41925-9 |
| Outcome | 24Hr Oxygen Device | Oxygen Device | Ventilation, High-flow/NIV, Low-flow, Room Air | - | 41925-9 |
| Outcome | 72Hr Oxygen Device | Oxygen Device | Ventilation, High-flow/NIV, Low-flow, Room Air | - | 41925-9 |
| Outcome | Death | - | - | - | - |
| Outcome | Time of Death | - | - | Hours | - |

**Extended Data** [**Table 2**](https://docs.google.com/document/d/1Wg4Qk0qxnWPjlK0IU5LicTAJ4wZjVCOAtr6BYasQ51U/edit?ts=5fc43130&pli=1#table_pcr) **|** Number of PCR positive and negative cases across the sites

| **Site** | **# Cases** | **# Pos. Cases** | **# Neg. Cases** | **% Pos. Cases** |
| --- | --- | --- | --- | --- |
| 1 | 2994 | 1057 | 1937 | 35.3% |
| 2 | 2825 | 139 | 2686 | 4.9% |
| 3 | 2697 | 258 | 2439 | 9.6% |
| 4 | 1786 | 618 | 1168 | 34.6% |
| 5 | 1065 | 347 | 718 | 32.6% |
| 6 | 853 | 427 | 426 | 50.1% |
| 7 | 724 | 168 | 556 | 23.2% |
| 8 | 637 | 232 | 405 | 36.4% |
| 9 | 565 | 342 | 223 | 60.5% |
| 10 | 485 | 304 | 181 | 62.7% |
| 11 | 400 | 400 | 0 | 100.0% |
| 12 | 346 | 346 | 0 | 100.0% |
| 13 | 213 | 114 | 99 | 53.5% |
| 14 | 176 | 72 | 104 | 40.9% |
| 15 | 102 | 102 | 0 | 100.0% |
| 16 | 99 | 99 | 0 | 100.0% |
| 17 | 74 | 49 | 25 | 66.2% |
| 18 | 55 | 55 | 0 | 100.0% |
| 19 | 28 | 28 | 0 | 100.0% |
| 20 | 24 | 15 | 9 | 62.5% |
| **Total** | **16148** | **5172** | **10976** | **32.0%** |

**Extended Data Table 3 | AUC for outcome prediction of patients from different ethnicities on the test set from 5 MGB sites**

| PredictionTime window | Task | Race | Local models (mean±std) | FL model | Improvement (%) |
| --- | --- | --- | --- | --- | --- |
| 24h | >=LFO | Black or African American | 0.880±0.030 | 0.926 | 5.15 |
|  |  | White or Caucasian | 0.836±0.025 | 0.885 | 5.85 |
|  | >=HFO/NIV | Black or African American | 0.875 ±0.047 | 0.968 | 10.59 |
|  |  | White or Caucasian | 0.862 ± 0.036 | 0.940 | 9.03 |
|  | >=MV | Black or African American | 0.869±0.053 | 0.957 | 10.08 |
|  |  | White or Caucasian | 0.873±0.031 | 0.963 | 10.40 |
| 72h | >=LFO | Black or African American | 0.839±0.034 | 0.919 | 6.93 |
|  |  | White or Caucasian | 0.782±0.033 | 0.847 | 8.26 |
|  | >=HFO/NIV | Black or African American | 0.836 ±0.042 | 0.907 | 8.47 |
|  |  | White or Caucasian | 0.809 ± 0.034 | 0.910 | 12.56 |
|  | >=MV | Black or African American | 0.821±0.056 | 0.897 | 9.31 |
|  |  | White or Caucasian | 0.795±0.038 | 0.935 | 17.63 |

**Extended Data** [**Table**](https://docs.google.com/document/d/1Wg4Qk0qxnWPjlK0IU5LicTAJ4wZjVCOAtr6BYasQ51U/edit?ts=5fc43130&pli=1#table_ethnic)**4 | p-values of ROC comparisons of local training to Federated Learning shown in Extended Data Fig. 5**

| **p-values** | **t>=0.25** | **t>=0.5** | **t>=0.75** |
| --- | --- | --- | --- |
| **Site 1** | 0.02674 | 0.03455 | 0.04015 |
| **Site 4** | 4.866e-05 | 1.08e-05 | 3.92e-05 |
| **Site 5** | 3.005e-06 | 5.098e-05 | 0.0001362 |
| **Site 6** | 1.717e-14 | 5.816e-08 | 2.357e-05 |
| **Site 8** | 2.872e-10 | 3.19e-06 | 5.872e-05 |
| **Site 12** | 0.08332 | 0.0507 | 0.02664 |

**Extended Data Algorithm 1 |** Client-server based federated learning (FL) using the FederatedAveraging algorithm ^7,16^ as implemented in NVIDIA Clara Train SDK^17^.


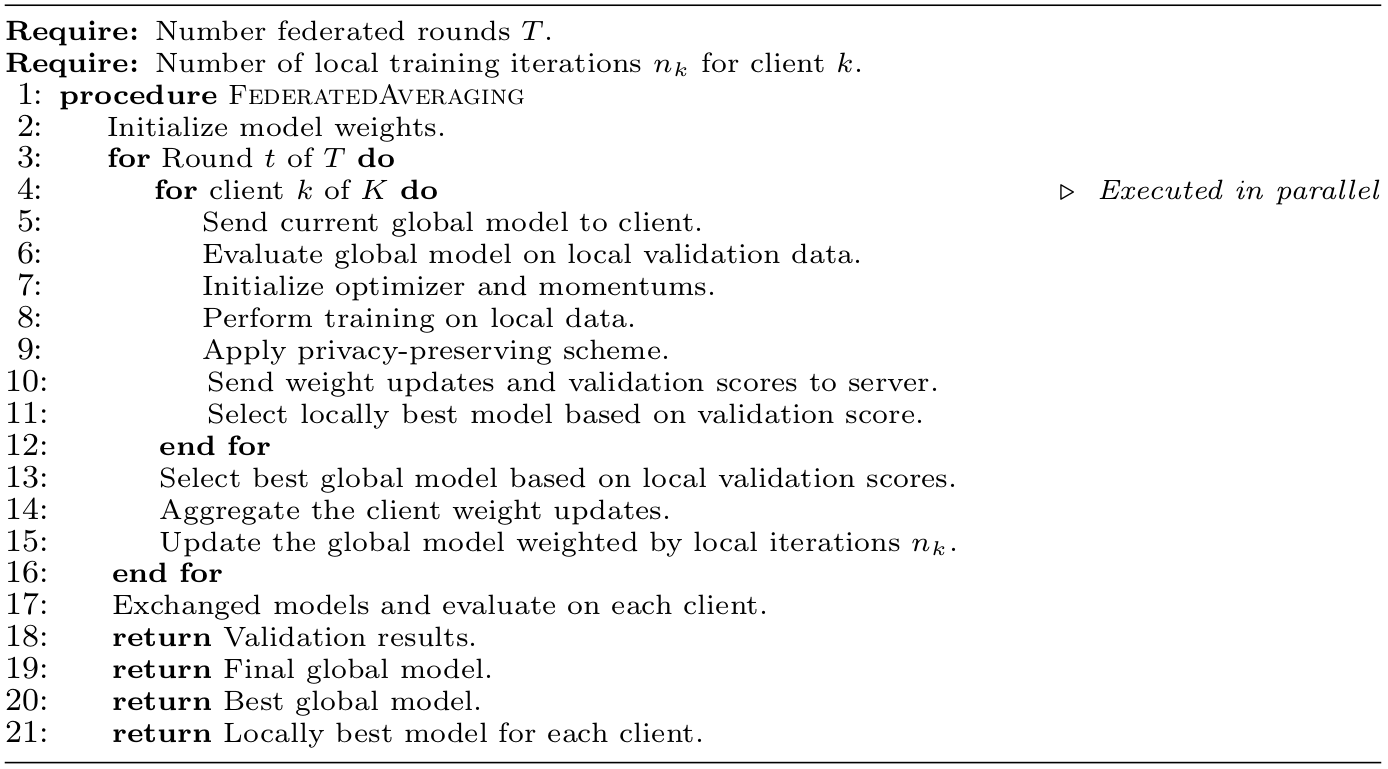

Supplement: Supplement [file eae5b8ddace7a81e128f85d3.docx]
